# Changes in Intraocular Pressure after Transepithelial Photorefractive Keratectomy and Femtosecond Laser In Situ Keratomileusis

**DOI:** 10.1155/2021/5592195

**Published:** 2021-03-10

**Authors:** Chien-Chih Chou, Po-Jen Shih, Hung-Chou Lin, Jun-Peng Chen, Jia-Yush Yen, I-Jong Wang

**Affiliations:** ^1^Department of Ophthalmology, Taichung Veterans General Hospital, Taichung, Taiwan; ^2^Institute of Clinical Medicine, College of Medicine, National Taiwan University, Taipei, Taiwan; ^3^Department of Biomedical Engineering, National Taiwan University, Taipei, Taiwan; ^4^Dr. Lin's Eye Clinic and Laser Vision Correction Center, Taoyuan, Taiwan; ^5^Biostatistics Task Force of Taichung Veterans General Hospital, Taichung, Taiwan; ^6^Department of Mechanical Engineering, National Taiwan University, Taipei, Taiwan; ^7^Department of Ophthalmology, National Taiwan University Hospital, Taipei, Taiwan

## Abstract

**Purpose:**

To investigate the changes in intraocular pressure (IOP) and biomechanically corrected IOP (bIOP) in patients undergoing transepithelial photorefractive keratectomy (TPRK) and femtosecond laser in situ keratomileusis (FS-LASIK) and to determine the effects of preoperative biomechanical factors on IOP and bIOP changes after FS-LASIK and TPRK.

**Design:**

A retrospective comparative study.

**Methods:**

We retrospectively investigated the IOP and corneal biomechanical changes in 93 eyes undergoing FS-LASIK and 104 eyes undergoing TPRK in a clinical setting. Preoperative and postoperative data on ophthalmic and Corvis ST examinations, in vivo Young's modulus, and noncontact tonometry were analyzed. Marginal linear regression models with generalized estimating equations were used for intragroup and intergroup comparisons of IOP and bIOP changes.

**Results:**

In the univariate model, IOP reduction after FS-LASIK was 2.49 mmHg higher than that after TPRK. In addition, bIOP reduction after FS-LASIK was 1.85 mmHg higher than that after TPRK. In the multiple regression model, we revealed that IOP reduction after FS-LASIK was 1.75 mmHg higher than that after TPRK. Additionally, bIOP reduction after FS-LASIK was 1.64 mmHg higher than that after TPRK. Postoperative changes in bIOP were less than those in IOP. In addition, Young's modulus and CBI had no significant effect on postoperative IOP and bIOP changes. We establish a biomechanically predictive model using the available data to predict postoperative IOP and bIOP changes after TPRK and FS-LASIK.

**Conclusions:**

Reductions in IOP and bIOP after FS-LASIK were 1.75 mmHg and 1.64 mmHg, respectively, more than those after TPRK, after adjustment for confounders. We revealed that the type of refractive surgery and peak distance (PD) were significant predictors of postoperative IOP and bIOP changes. By contrast, depth of ablation showed a significant effect on only IOP changes.

## 1. Introduction

Corneal refractive surgery changes the central corneal thickness (CCT) and biomechanical properties, including corneal curvature and refractive power [1–3]. Laser in situ keratomileusis (LASIK) is the most widely used corneal refractive surgical procedure for myopia and astigmatism correction [4]. It involves the creation of a corneal flap using a microkeratome or femtosecond laser, which is lifted to expose the corneal stroma for excimer laser ablation [4, 5]. Consequently, flap dissection and CCT reduction alter the corneal shape and biomechanical properties [6]. Accordingly, LASIK has been associated with the development of corneal ectasia and underdiagnosis of glaucoma or undetected glaucoma progression due to the underestimation of intraocular pressure (IOP) [7, 8]. By contrast, photorefractive keratectomy (PRK) is performed using an excimer laser directly irradiated to the corneal stroma after removal of the corneal epithelium using scrapping, alcohol, or an excimer laser [9]. Unlike LASIK, the incidence of ectasia following PRK is considered extremely rare [10]. However, IOP underestimation was also noted after PRK [11]. Recently, transepithelial photorefractive keratectomy (TPRK) was reintroduced to minimize the complications associated with conventional PRK and LASIK, especially postoperative corneal biomechanical stability [12]. Therefore, TPRK is often considered in patients who wish to undergo LASIK and who have a risk of post-LASIK corneal biomechanical instability [13].

Several advanced parameters have been developed to measure corneal biomechanical properties and the corrected IOP after refractive surgery. Biomechanically corrected IOP (bIOP) and Corvis Biomechanical Index (CBI) can be estimated using the noncontact tonometer Corvis ST (Oculus, Wetzlar, Germany). bIOP was developed using the finite element simulations applied to the human eye model and to compensate for variations in thickness and material stiffness in algorithm [11, 14–16]. The CBI was developed by Vinciguerra et al. to evaluate corneal biomechanical properties [17, 18].

Other previous parameters from Corvis ST indices, such as the deformation amplitude (DA), the first applanation time (A1T), and the first applanation velocity (A1V), have also been used to measure corneal biomechanical properties [19]. These indices help detect abnormal corneal morphology, such as keratoconus. However, differentiating the subclinical keratoconus from astigmatism before refractive surgery by using these indices is not sufficient because these are affected by the corneal thickness, IOP, and corneal geometry [13, 20, 21].

In our previous studies, we obtained images using Corvis ST to derive an in vivo Young's modulus, a commonly used mechanical property, which is believed to be more feasible and more applicable for future clinical applications and to provide more productive implications for refractive surgery [22–24]. Furthermore, Young's modulus represents material stiffness, is independent of the corneal thickness, and can help detect keratoconus [25] and post-LASIK ectasia [26, 27]. Therefore, it is critical to study the effects of in vivo Young's modulus and CBI on IOP and bIOP changes after FS-LASIK and TPRK in terms of corneal biomechanical properties. In the current study, we investigated the effects of the CBI, in vivo Young's modulus, and other dynamic corneal response parameters from the Corvis ST on IOP and bIOP changes after FS-LASIK and TPRK with marginal linear regression models using generalized estimating equations (GEEs).

## 2. Materials and Methods

We retrospectively investigated medical records of 114 enrolled patients from Dr Lin's Clinics between March 2012 and December 2019. Patients who did not return for the 1-month follow-up were excluded. Other exclusion criteria were previous ocular surgery; concomitant diseases such as glaucoma, uveitis, corneal ectatic disease, Fuchs' dystrophy, diabetic retinopathy, and systemic collagen diseases; chronic use of topical ophthalmic medications; corneal scars or opacities; and irregular astigmatism. A quality score was calculated after the Corvis ST measurement; measurements with poor quality scores (model deviation) were excluded from the statistical analyses. The choice of surgical procedure mainly depended on the patient's preference, fear of ectasia, and dry eye severity. Of the 114 patients in the study, 54 had FS-LASIK and 60 had TPRK. Most of them have the procedure done on both eyes. The research followed the tenets of the Declaration of Helsinki and was approved by the Institutional Review Board of National Taiwan University Hospital. For retrospective chart reviews, a waiver of consent was approved by the Institutional Review Board.

### 2.1. Surgical Techniques

#### 2.1.1. FS-LASIK

An experienced surgeon performed all FS-LASIK procedures after administering topical anesthesia. All patients were treated using an excimer laser and FS-LASIK flap technique on the same day. A WaveLight Allegretto Wave Eye-Q laser (Alcon Laboratories, Fort Worth, TX, USA) with flying spot technology of a 0.68 mm full-width-half-maximum, a repetition rate of 400 Hz, and an eye tracker of 400 Hz were used. For flap creation, an LDV femtosecond laser (Ziemer, Port, Switzerland) was used in all cases. A 9.0 mm flap with a 5 mm superior hinge was cut in each case. The flap thickness was predetermined at 100 *μ*m in all cases. The ablation zone selected had a size of 6.1–8.0 mm. The excimer laser ablation was centered on the corneal vertex. All aspheric treatments were prepared using the wavefront-optimized mode (Alcon Laboratories). Fluorometholone 0.02% and levofloxacin 0.5% were applied 4 times a day for 3 days. Postoperative follow-ups were routinely performed after 1 day, 1 week, and 1 month in all cases.

#### 2.1.2. TPRK

Photoablation was performed first using the phototherapeutic keratectomy program and then a wavefront-optimized program with the WaveLight Allegretto Wave Eye-Q laser platform (Alcon Laboratories). WaveLight Allegretto's company-supplied nomogram was used for all patients, and the ablation zone selected had a size of 5.0–6.0 mm. Postoperatively, 1 drop of topical levofloxacin 0.5% was instilled at the surgical site and a bandage contact lens (Focus Dailies, CIBA vision) was placed on the cornea and then removed 2 to 3 days later after the corneal epithelium was completely healed. Topical levofloxacin 0.5% was applied 4 times per day for 3 days. Fluorometholone 0.02% was applied 4 times per day for more than 1 month and then tapered over 3 months.

### 2.2. Outcome Measures

The images and data of eyes that underwent FS-LASIK and TPRK were used for analysis. Each clinical examination involved tests for visual acuity, slit-lamp microscopy of the anterior and posterior segments, corneal topography, CCT (measured using a handy pachymeter [SP-100; Tomey, Nagoya, Japan]), and Corvis ST measurement. Preoperative variables were age, sex, corneal spherical equivalent, depth of ablation, central corneal keratometry values (measured using Topcon KR 800; Tokyo, Japan), IOP, and flap thickness. Flap thickness was recorded as the intended flap setting in the surgery. Data of postoperative follow-up at 1 day, 1 week, and 1 month were considered.

Corvis ST measurements were performed as previously described [24, 28]. Corvis ST provides in vivo deformation during air puff with maximal pressure at 60 mmHg [29]. DCR parameters included CCT, A1T, A1L, A1V, DA, peak distance (PD), radius, A2T, A2L, A2V, DA ratio 2.0 mm, IntInvRad, Ambrósio's relational thickness (ARTh), and stiffness parameter-A1 (SP-A1). (Definitions and descriptions of the parameters are provided in the following paragraphs.) Briefly, the recording starts with the cornea at the natural convex shape. The air puff forces the cornea inward (i.e., the ingoing phase) by using applanation (i.e., the first or ingoing applanation) into a concavity phase until it achieves the highest concavity (HC). An oscillation period precedes the outgoing or returning phase. The cornea then undergoes a second applanation before achieving its natural shape, with possible oscillation. The first applanation time (A1T) and the second applanation time (A2T) are the length of time from the initiation of the air puff to the first and the second applanation. A1L and A2L (A length: −1/−2) are the length of the flattened cornea at the first and second applanations, respectively. A1V and A2V (velocity: −1/−2) are the corneal velocity during the first and second applanations, respectively. HC time is the length of time from the start of deformation to the point when the cornea reaches the HC. Radius (HC curvature) is the central curvature radius at the HC. The PD is the distance between the 2 surrounding peaks of the cornea at the HC. DA is the movement of the corneal apex from the start of deformation to the HC. The movement of the corneal apex is compensated by the movement of the whole eye. Hence, only the movement of the cornea is described by this parameter. The IOP measured using Corvis ST can be derived from the dynamics of cornea deformation indented by an air puff. IOP is calculated based on the timing of the first applanation event [15, 30, 31]. CCT is also calculated using the horizontal Scheimpflug image. The lowest value is displayed.

The Vinciguerra Screening Report is a new display of the Corvis ST aimed to report the comparison of normative values and to include an index to separate normal from keratoconic patients [32]. The Corvis ST provides a bIOP value, which offers an estimation of true IOP. New dynamic corneal response parameters in the Vinciguerra Screening Report provides the DA ratio of 2.0 mm, IntInvRad, ARTh, and SP-A1. The DA ratio of 2.0 mm represents the ratio between the DA of the apex and the average of 2 points located 2.0 mm on either side of the apex, with a larger value indicating lower corneal resistance to deformation. The IntInvRad parameter was the reciprocal of the radius of curvature at the highest concavity. A higher IntInvRad indicates greater corneal compliance [11]. Corvis ST enables the calculation of a new corneal thickness index, ARTh, by characterizing the thickness data on the horizontal Scheimpflug image, with a lower value indicating a faster thickness increase toward the periphery [17]. The SP-A1 is calculated as resultant pressure divided by displacement. The resultant pressure is calculated as the adjusted pressure at A1 minus the biomechanically corrected IOP. The displacement is the distance the corneal apex moves from the predeformation state to A1. A larger SP-A1 value indicates a stiffer cornea [17, 33]. The CBI is an intuitive method to evaluate the probability of corneal ectasia [17]. The CBI is calculated using DA ratio max 1 mm, DA ratio max 2 mm, A1V, the standard deviation of deformation amplitude at highest concavity, ARTh, and SP-A1 by using logistic regression [17]. A CBI of ≥0.5 indicates possible keratoconic eyes. Hence, CBI < 0.5 is categorized as “low risk,” and CBI ≥0.5, as “high risk” [17]. Moreover, we used a closed-form solution to perform a rapid estimation of the corneal biomechanical properties during the air puff. The model is based on the static fluid-filled hemispherical shell model subjected to a concentrated load. This proposed model can directly provide in vivo Young's modulus as opposed to the various parameters defined by Corvis ST [24].

### 2.3. Statistical Analysis

The unit of analysis was the eye. Data for measurements before and 1 month after FS-LASIK and TPRK are expressed as medians and interquartile ranges. Statistical analysis was performed using SPSS (v22.0; IBM, New York, USA). The nonparametric Wilcoxon signed-rank test was used to compare IOP and bIOP before and after surgery. The nonparametric Mann–Whitney *U* test was used to compare IOP, bIOP, and Corvis ST measurement parameters between FS-LASIK and TPRK. The nonparametric Spearman's correlation coefficient was used to assess the association between IOP, bIOP, and corneal biomechanical parameters obtained using Corvis ST.

To account for the intereye correlation of a patient and increased power and precision, data were analyzed with marginal linear regression models using the GEE to evaluate the factors affecting the changes in IOP and bIOP [34]. We used a robust estimator for the covariance matrix and an exchangeable correlation structure for the working correlation matrix. In the multivariate model, the statistically significant factors in the univariate model were selected. We excluded A1T and A2T because they were involved in the calculation and standard definition of IOP [15]. To avoid multicollinearity, the correlations between variables were analyzed. We removed some of the highly correlated variables listed in Supplementary Table S1 and included only the type of surgery, A1V, PD, CBI, IntInvRad, and depth of ablation. Estimated *β* values with 95% CIs were calculated for all the parameters. All *P* values were 2-sided and considered statistically significant at <05.

## 3. Results

**Figure 1:**
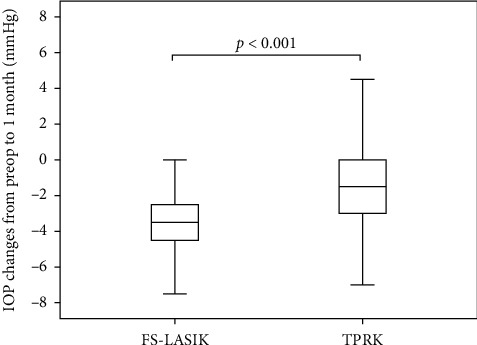
Box plot of intraocular pressure (IOP) changes from before surgery to 1 month after transepithelial photorefractive keratectomy (TPRK) and femtosecond laser in situ keratomileusis (FS-LASIK). The bottom and top of each box represent the lower and upper quartiles, respectively. The line inside each box represents the median. The bars represent data within 1.5 times the interquartile range. Reductions in IOP after FS-LASIK were 1.75 mmHg more than those after TPRK, after adjusting for confounders. *P* < 0.001.

**Figure 2:**
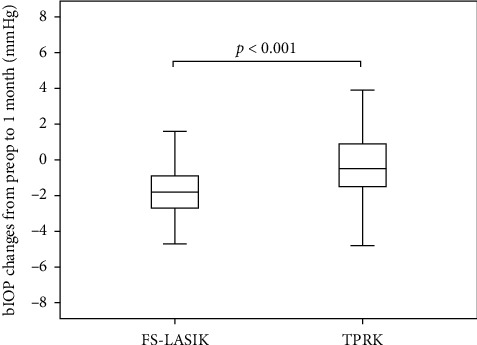
Box plot of biomechanically corrected intraocular pressure (bIOP) changes from before surgery to 1 month after transepithelial photorefractive keratectomy (TPRK) and femtosecond laser in situ keratomileusis (FS-LASIK). The bottom and top of each box represent the lower and upper quartiles, respectively. The line inside each box represents the median. The bars represent data within 1.5 times the interquartile range. Reductions in bIOP after FS-LASIK were 1.64 mmHg more than those after TPRK, after adjusting for confounders.

We enrolled 54 patients in the FS-LASIK group and 60 patients in the TPRK group. Most of them have the procedure done on both eyes. We analyzed 93 eyes underwent FS-LASIK and 104 eyes underwent TPRK.  Table 1 presents the characteristics of both groups. The following baseline characteristics were significantly different between the 2 groups: depth of ablation, IOP, bIOP, A1L, A1V, A2L, A2V, PD, DA, CCT, A1T, radius, Young's modulus, CBI, integrated radius, DA ratio, ARTh, and SP-A1. IOP and bIOP changed significantly less after TPRK than after FS-LASIK (Figures 1 and 2).

The nonparametric Spearman's correlation method was used to identify potential factors associated with changes in IOP and bIOP after FS-LASIK and TPRK (Table 2). In the FS-LASIK group, IOP was significantly correlated with SE, depth of ablation, A1V, DA, CCT, PD, A1T, A2T, CBI, DA ratio 2.0 mm, IntInvRad, ARTh, SP-A1, and preoperative IOP; also, bIOP was significantly correlated with A1T, DA ratio 2.0 mm, and preoperative bIOP (Table 2). In the TPRK group, IOP was significantly correlated with SE, depth of ablation, A1L, A1T, A2V, A1T, and preoperative IOP; also, bIOP was significantly correlated with A1L, A2V, CCT, DA ratio 2.0 mm, and preoperative bIOP (Table 2). Young's modulus was not correlated with IOP and bIOP changes in either group.

In the univariate regression analysis with GEE, we analyze postoperative IOP and bIOP changes (Tables 3 and 4 , respectively). IOP reduction after FS-LASIK was 2.49 mmHg higher than that after TPRK. In addition, bIOP reduction after FS-LASIK was 1.85 mmHg higher than that after TPRK. We also found that SE, A1V, PD, DA, A1T, A2T, IntInvRad, SP-A1, and preoperative IOP had significant effects on predicting changes in both IOP and bIOP. By contrast, depth of ablation and CBI only exerted significant effects on predicting IOP changes.

In the multiple regression analysis with GEE, we revealed that refractive procedures (FS-LASIK or TPRK), depth of ablation, and PD had significant effects on predicting postoperative IOP changes (*P* < 0.01, *P* < 0.01, and *P*=0.004, respectively; Table 3). After adjustment for confounders, IOP reduction after FS-LASIK was 1.75 mmHg higher than that after TPRK. Regarding bIOP, we found that refractive procedures (FS-LASIK or TPRK) and PD had significant effects on predicting postoperative bIOP changes (*P* < 0.01 and *P*=0.004, respectively; Table 4). After adjustment for confounders, bIOP reduction after FS-LASIK was 1.64 mmHg higher than that after TPRK. However, Young's modulus and CBI had no significant effect on postoperative IOP and bIOP changes. Furthermore, we developed a biomechanically predictive model to predict postoperative IOP and bIOP changes after TPRK and FS-LASIK (Tables 3 and 4).

## 4. Discussion

In the current study, IOP reduction after FS-LASIK was 2.49 mmHg higher than that after TPRK. After adjustment for confounders in the marginal linear regression model, IOP reduction after FS-LASIK was 1.75 mmHg higher than that after TPRK. This finding is consistent with previous studies [35–37]. Lee et al. demonstrated that IOP was underestimated by 1.5 mmHg for TPRK and 3.5 mmHg for FS-LASIK, respectively [35]. Chang et al. revealed a decrease of 1.36 mmHg by extrapolating their data to a theoretical correction of zero diopters after LASIK [36]. Schallhorn et al. likewise estimated a decrease of 0.94 mmHg resulting from corneal flap after LASIK [37]. All of them reasoned that the higher underestimation of IOP in FS-LASIK is due to flap dissection.

In the present study, the reduction in bIOP after FS-LASIK was 1.85 mmHg and 1.64 mmHg higher than that after TPRK, respectively, before and after correction of confounders. By contrast, studies have demonstrated that the bIOP was stable both after PRK and LASIK [11, 15]. This difference may be explained by the different statistical methods used. We used the GEE approach to maximize precision and account for intereye correlation [38]. Our finding implies that bIOP is affected by the corneal structure and biomechanical changes from the FS-LASIK flap dissection.

We found an IOP decrease of 0.24 mmHg (95% CI, 0.06–0.43 mmHg) per diopter of myopic correction in our marginal linear regression model. Schallhorn et al. reported a decrease of 0.40 mmHg (95% CI, 0.39–0.41 mmHg) per diopter of myopic correction for both PRK and LASIK [37]. Similarly, a decrease of 0.03 mmHg (95% CI, 0.02–0.04 mmHg) per micrometer of depth of ablation was found in our model, which is close to the value of 0.032 mmHg per micrometer of depth of ablation determined by Schallhorn et al. for PRK and LASIK [37]. After adjustment for confounders, depth of ablation contributed to 0.02 mmHg (95% CI, 0.01–0.03 mmHg) of IOP change per micrometer in our study. However, we also found that the diopter correction and the depth of ablation did not affect the change in postoperative bIOP for either FS-LASIK or TPRK. These results imply that bIOP is independent of the effect of depth of ablation, in terms of the definition and calculation of bIOP [32].

We found that the preoperative IOP affected postoperative IOP changes (*P* < 0.001)—in particular, a higher preoperative IOP led to a higher postoperative decrease in IOP, consistent with Schallhorn et al. [37]. Similarly, higher preoperative bIOP led to a higher postoperative decrease in bIOP (*P* < 0.001). In addition, Schallhorn et al. reported that preoperative CCT was associated with the magnitude of the IOP change after refractive surgery: 0.0097 mmHg/µm in preoperative CCT for LASIK and 0.0096 mmHg/µm in preoperative CCT for PRK [37]. In our study, the IOP change was 0.01 mmHg/µm (*P*=0.122) in preoperative CCT after adjustment for confounders. This intriguing finding suggested that preoperative CCT was an independent predictor of the amount of postoperative IOP decrease. In other words, thinner corneas experienced more IOP changes after refractive surgery and thus were more vulnerable to ablation. By contrast, in our model, preoperative CCT was not associated with postoperative bIOP changes (*P*=0.727). A previous study revealed that bIOP is a close estimate of true IOP [16]. bIOP is an index developed to be independent of the effect of corneal biomechanical properties. We also found that bIOP was less affected by refractive surgery. Thus, bIOP change is less affected by preoperative CCT.

Our results revealed that PD was a significant predictor of postoperative IOP and bIOP changes, which has not been discussed before. PD has a negative relationship with stiffness and overall structure resistance [39]. We reasoned that corneas with higher PD are vulnerable to ablation. The ablation in FS-LASIK and TPRK weakens corneal biomechanical structures, causing a larger bending area and PD and, thus, a greater magnitude of postoperative IOP and bIOP decrease.

Our results also indicated that DA was a significant predictor of postoperative IOP and bIOP changes, which has not been mentioned before. DA is incited by the uniaxial air puff, which causes a constant compression of the extracellular matrix and extension of parts of the collagen lamellae [40]. DA is an interaction and performance among geometric structures and biomechanical properties [41–43]. Therefore, larger DA implied weaker cornea. Consequently, weaker corneal structural resistance after refractive surgery leads to more biomechanical alternations; this explains why postoperative IOP and bIOP changes are larger when DA is higher.

Young's modulus represents the elastic properties of a material and is the ratio of stress to strain [44]. In the present study, Young's modulus was not a significant predictor of postoperative IOP and bIOP changes. Young's modulus represents the corneal material property instead of the overall corneal structural resistance to the air puff [24]. Refractive surgery causes more disruption in the overall structural resistance than in the material property; this explains why the magnitude of postoperative IOP change was not affected by Young's modulus.

No study has investigated the effect of the CBI on the postoperative changes in IOP and bIOP. The CBI was developed to evaluate corneal biomechanical properties and the probability of corneal ectasia, and a CBI of ≥0.5 indicates a higher possibility of corneal ectasia [17]. A study evaluated the CBI to detect ectasia in 312 patients with healthy cornea and 118 patients with keratoconus (asymmetric ectasia). The area under the ROC curve was 0.864, and the sensitivity and specificity values were 70.7% and 93.3%, respectively [18]. Our study revealed that the CBI was not a significant predictor of postoperative IOP and bIOP changes after adjustment for confounders. This may be because the CBI, developed to standardize the available biomechanical parameters, indicates the probability of corneal ectasia in terms of the geometric changes of corneal profile and not IOP [17, 45].

Many factors have been proposed to predict changes in IOP and bIOP estimation after LASIK and PRK, including preoperative IOP, CCT, corneal curvature, SE, and depth of ablation [37, 46]. Compared with previous models [37, 46], our models further provided the corneal biomechanical properties that affect postoperative IOP and bIOP estimation. We analyzed these factors after adjusting for confounding variables. Furthermore, our use of marginal modeling with GEE allows intereye correlation and maximize precision [38]. This approach can adequately minimize the confounding effects.

This study has some limitations. First, the longer duration of postoperative topical steroids treatment in the TPRK group, which may affect IOP measurements. However, low-concentration fluorometholone 0.02% in our study is less likely to elevate IOP according to previous studies. Second, 1 month may be a relatively short follow-up duration. However, a study revealed that the IOP was stable during between the 1-month and 3-month follow-up visits after PRK and LASIK [37]. Third, our sample size was relatively small. Fourth, determining the effect of diurnal IOP changes is difficult. Therefore, data regarding the diurnal variation of IOP were not included. Additional studies based on a larger sample size and for a longer follow-up period are required to determine the underlying pathophysiology.

## 5. Conclusion

Reductions in IOP and bIOP after FS-LASIK were 1.75 mmHg and 1.64 mmHg, respectively, more than those after TPRK, after adjustment for confounders. We demonstrated that the type of refractive surgery and PD were significant predictors of postoperative IOP and bIOP changes. By contrast, depth of ablation showed a significant effect on only IOP changes.

## Figures and Tables

**Table 1:** Patients' demographic data, refractive status, and corneal biomechanical properties.

	FS-LASIK (*n* = 93)	TPRK (*n* = 104)	*P* value†
Gender, %			0.409
Male	31 (33.3%)	28 (26.9%)	
Female	62 (66.7%)	76 (73.1%)	
Age (year)	32.00 (25.00, 37.00)	32.50 (26.00, 40.00)	0.549
SE (D)	−5.75 (−6.81, −4.50)	−5.49 (−7.00, −3.87)	0.768
Average K (*D*)	43.50 (42.61, 44.87)	43.56 (42.77, 44.48)	0.943
Depth of ablation (*μ*m)	68.00 (53.00, 84.50)	47.50 (25.50, 66.75)	<0.001*∗∗*
IOP (mmHg)	14.50 (13.50, 16.00)	13.50 (12.50, 14.50)	<0.001*∗∗*
bIOP (mmHg)	14.60 (13.65, 15.70)	13.95 (12.90, 15.18)	<0.004*∗∗*
CCT (*μ*m)	530 (518, 557)	513 (502, 532)	<0.001*∗∗*
A1L (mm)	2.29 (1.89, 2.53)	1.91 (1.86, 2.13)	<0.001*∗∗*
A1V (m/s)	0.15 (0.14, 0.16)	0.16 (0.15, 0.17)	<0.001*∗∗*
A2L (mm)	1.96 (1.80, 2.05)	1.87 (1.49, 2.02)	<0.004*∗∗*
A2V (m/s)	−0.28 (−0.29, −0.26)	−0.30 (−0.31, −0.28)	<0.001*∗∗*
A1T (ms)	7.43 (7.29, 7.62)	7.12 (7.01, 7.27)	<0.001*∗∗*
A2T (ms)	21.95 (21.78, 22.11)	22.06 (21.82, 22.32)	0.013*∗*
Radius (mm)	7.11 (6.69, 7.71)	6.72 (6.41, 7.24)	<0.001*∗∗*
PD (mm)	5.11 (4.98, 5.25)	5.22 (5.01, 5.40)	<0.001*∗∗*
DA (mm)	1.07 (1.01, 1.11)	1.14 (1.07, 1.21)	<0.001*∗∗*
Young's modulus (MPa)	0.45 (0.38, 0.56)	0.64 (0.46, 0.89)	<0.001*∗∗*
CBI	0.30 (0.05, 0.74)	0.78 (0.44, 0.96)	<0.001*∗∗*
DA ratio	4.70 (4.40, 5.05)	5.10 (4.90, 5.50)	<0.001*∗∗*
Integrated radius (mm^−1^)	8.50 (7.70, 9.20)	9.10 (8.70, 9.70)	<0.001*∗∗*
ARTh	421.30 (366.80, 469.65)	385.85 (343.28, 445.25)	<0.011*∗*
SP-A1	93.90 (83.85, 101.55)	82.90 (76.60, 88.98)	<0.001*∗∗*

Mann–Whitney *U* test, median (interquartile range). *∗P* < 0.05 and *∗∗P* < 0.01. FS-LASIK, femtosecond laser in situ keratomileusis; TPRK, transepithelial photorefractive keratectomy; SE, spherical equivalent; K, keratometric readings in diopter; IOP, intraocular pressure; bIOP, biomechanically corrected IOP; A1L, first applanation length; A1V, applanation-1 velocity; A2L, second applanation length; A2V, applanation-2 velocity; PD, peak distance; DA, deformation amplitude; CCT, central corneal thickness; A1T, applanation-1 time; A2T, applanation-2 time; CBI, Corvis Biomechanical Index; DA ratio, DA ratio at 2.0 mm; Integrated radius, the integrated area under the radius of the inversed curvature during the concave phase; ARTh, Ambrósio's relational thickness in the horizontal profile; SP-A1, stiffness parameter at applanation 1. † LASIK vs PR.

**Table 2:** Potential factors associated with changes in IOP and bIOP after FS-LASIK and TPRK.

	LASIK (*N* = 93)		PRK (*N* = 104)	
	∆IOP	∆bIOP	∆IOP	∆bIOP
SE (D)	0.225*∗*	0.073	0.254*∗∗*	0.085
Average K (D)	0.099	0.047	−0.182	−0.162
Depth of ablation (*μ*m)	−0.426*∗∗*	−0.146	−0.278*∗∗*	−0.029
IOP preop (mmHg)	−0.423*∗∗*	--	−0.194*∗*	--
bIOP preop (mmHg)	--	−0.263*∗*	--	−0.240*∗*
CCT (*μ*m)	−0.310*∗∗*	−0.063	0.129	0.271*∗∗*
A1L (mm)	−0.139	−0.093	0.199*∗*	0.231*∗*
A1V (m/s)	0.293*∗∗*	0.190	−0.067	−0.113
A2L (mm)	−0.025	−0.031	0.002	0.034
A2V (m/s)	−0.088	−0.140	−0.213*∗*	−0.200*∗*
A1T (ms)	−0.435*∗∗*	−0.279*∗∗*	−0.211*∗*	−0.169
A2T (ms)	0.205*∗*	0.094	0.117	0.058
Radius (mm)	−0.084	−0.042	0.107	0.112
PD (mm)	0.178	0.082	0.152	0.112
DA (mm)	0.215*∗*	0.137	0.187	0.154
Young's modulus (MPa)	0.019	0.015	−0.113	−0.116
CBI	0.330*∗∗*	0.169	0.063	−0.031
DA ratio	0.400*∗∗*	0.278*∗∗*	−0.124	−0.198*∗*
Integr. radius (mm^−1^)	0.284*∗∗*	0.148	−0.038	−0.075
ARTh	−0.193	−0.087	−0.061	−0.011
SP-A1	−0.324*∗∗*	−0.108	0.010	0.110

Correlation coefficient. *∗P* < 0.05 and *∗∗P* < 0.01. FS-LASIK, femtosecond laser in situ keratomileusis; TPRK, transepithelial photorefractive keratectomy; SE, spherical equivalent; K, keratometric readings in diopter; IOP, intraocular pressure; bIOP, biomechanically corrected IOP; A1L, first applanation length; A1V, applanation-1 velocity; A2L, second applanation length; A2V, applanation-2 velocity; PD, peak distance; DA, deformation amplitude; CCT, central corneal thickness; A1T, applanation-1 time; A2T, applanation-2 time; CBI, Corvis Biomechanical Index; DA ratio, DA ratio at 2.0 mm; Integr. Radius, the integrated area under the radius of the inversed curvature during the concave phase; ARTh, Ambrósio's relational thickness in the horizontal profile; SP-A1, stiffness parameter at applanation 1.

**Table 3:** Potential factors affecting the changes in IOP after FS-LASIK and TPRK were analyzed with marginal linear regression models using the GEE.

	Univariate			*P* value	Multivariate			*P* value
	B	SE	95% CI		B	SE	95% CI	
Group								
LASIK	Ref.				Ref.			
PRK	2.49	(0.40)	(1.71, 3.27)	<0.001*∗∗*	1.75	(0.49)	(0.79, 2.70)	<0.001*∗∗*
Gender								
Male	Ref.							
Female	−0.17	(0.53)	(−1.22, 0.87)	0.745				
Age (year)	−0.02	(0.03)	(−0.07, 0.04)	0.590				
SE (D)	0.24	(0.09)	(0.06, 0.43)	0.009*∗∗*				
Average K (D)	0.01	(0.20)	(−0.38, 0.40)	0.957				
Depth of ablation (*μ*m)	−0.03	(0.01)	(−0.04, −0.02)	<0.001*∗∗*	−0.02	(0.01)	(−0.03, −0.01)	0.004*∗∗*
IOP preop	−0.69	(0.08)	(−0.84, −0.54)	<0.001*∗∗*				
CCT (*μ*m)	−0.01	(0.01)	(−0.03, 0.00)	0.122				
A1L (mm)	−0.55	(0.51)	(−1.55, 0.45)	0.279				
A1V (m/s)	28.61	(8.69)	(11.58, 45.64)	0.001*∗∗*	−5.57	(11.05)	(−27.23, 16.10)	0.615
A2L (mm)	−0.68	(0.38)	(−1.42, 0.06)	0.071				
A2V (m/s)	−13.29	(8.68)	(−30.30, 3.72)	0.126				
A1T (ms)	−4.79	(0.52)	(−5.82, −3.76)	<0.001*∗∗*				
A2T (ms)	2.11	(0.56)	(1.01, 3.20)	<0.001*∗∗*				
Radius (mm)	−0.07	(0.17)	(−0.39, 0.26)	0.697				
PD (mm)	3.80	(0.74)	(2.35, 5.25)	<0.001*∗∗*	2.79	(0.84)	(1.15, 4.44)	<0.001*∗∗*
DA (mm)	9.89	(1.73)	(6.50, 13.27)	<0.001*∗∗*				
Young's modulus								
(MPa)	0.15	(0.21)	(−0.26, 0.56)	0.462				
CBI	1.10	(0.41)	(0.29, 1.91)	0.008*∗∗*	0.15	(0.39)	(−0.62, 0.92)	0.706
DA ratio	0.71	(0.45)	(−0.17, 1.59)	0.112				
Integr. radius (mm^−1^)	0.53	(0.18)	(0.18, 0.88)	0.003*∗∗*	0.09	(0.23)	(−0.37, 0.55)	0.692
ARTh	0.00	(0.00)	(−0.00, 0.00)	0.299				
SP-A1	−0.04	(0.02)	(−0.08, −0.01)	0.015*∗*				

∆IOP = −16.47 + 1.75 (PRK) − 5.57 (A1V) + 2.79 (peak distance) + 0.15 (CBI) + 0.09 (Integr. radius) − 0.02 (ablation depth). GEE, generalized estimating equation. *∗P* < 0.05 and *∗∗P* < 0.01. FS-LASIK, femtosecond laser in situ keratomileusis; TPRK, transepithelial photorefractive keratectomy; SE, spherical equivalent; K, keratometric readings in diopter; IOP, intraocular pressure; bIOP, biomechanically corrected IOP; A1L, first applanation length; A1V, applanation-1 velocity; A2L, second applanation length; A2V, applanation-2 velocity; PD, peak distance; DA, deformation amplitude; CCT, central corneal thickness; A1T, applanation-1 time; A2T, applanation-2 time; CBI, Corvis Biomechanical Index; DA ratio, DA ratio at 2.0 mm; Integr. radius, the integrated area under the radius of the inversed curvature during the concave phase; ARTh, Ambrósio's relational thickness in the horizontal profile; SP-A1, stiffness parameter at applanation 1.

**Table 4:** Potential factors affecting the changes in bIOP after FS-LASIK and TPRK were analyzed with marginal linear regression models using the GEE.

	Univariate			*P* value	Multivariate			*P* value
	B	SE	95% CI		B	SE	95% CI	
Group								
LASIK	Ref.				Ref.			
PRK	1.85	(0.37)	(1.13, 2.57)	<0.001*∗∗*	1.64	(0.47)	(0.72, 2.57)	0.001*∗∗*
Gender								
Male	Ref.							
Female	−0.42	(0.47)	(−1.35, 0.50)	0.370				
Age (year)	−0.02	(0.02)	(−0.07, 0.03)	0.371				
SE (D)	0.07	(0.09)	(−0.10, 0.24)	0.409				
Average K (D)	0.01	(0.17)	(−0.33, 0.35)	0.950				
Depth of ablation (*μ*m)	−0.01	(0.01)	(−0.02, 0.00)	<0.109	0.002	(0.01)	(−0.01, 0.01)	0.702
bIOP preop (mmHg)	−0.63	(0.10)	(−0.83, −0.43)	<0.001*∗∗*				
CCT (*μ*m)	0.00	(0.01)	(−0.01, 0.02)	0.727				
A1L (mm)	−0.27	(0.48)	(−1.21, 0.66)	0.567				
A1V (m/s)	18.54	(7.99)	(2.87, 34.21)	0.020*∗*	−4.20	(10.60)	(−24.97, 16.57)	0.692
A2L (mm)	−0.63	(0.34)	(−1.30, 0.03)	0.061				
A2V (m/s)	−11.02	(7.87)	(−26.44, 4.40)	0.161				
A1T (ms)	−3.59	(0.52)	(−4.61, −2.57)	<0.001*∗∗*				
A2T (ms)	1.63	(0.52)	(0.61, 2.65)	<0.002*∗∗*				
Radius (mm)	−0.02	(0.14)	(−0.30, 0.26)	0.876				
PD (mm)	2.96	(0.69)	(1.61, 4.32)	<0.001*∗∗*	2.35	(0.81)	(0.77, 3.94)	0.004*∗∗*
DA (mm)	7.61	(1.60)	(4.47, 10.76)	<0.001*∗∗*				
Young's modulus (MPa)	0.13	(0.18)	(−0.23, 0.49)	0.469				
CBI	0.69	(0.35)	(0.00, 1.38)	0.050	0.09	(0.35)	(−0.60, 0.78)	0.798
DA ratio	0.42	(0.31)	(−0.18, 1.02)	0.167				
Integr. radius (mm^−1^)	0.32	(0.15)	(0.03, 0.61)	0.029*∗∗*	0.001	(0.22)	(−0.43, 0.43)	0.997
ARTh	0.00	(0.00)	(−0.00, 0.00)	0.699				
SP-A1	−0.02	(0.02)	(−0.05, 0.01)	0.169*∗*				

∆bIOP = −13.39 + 1.64 (PRK) − 4.20 (A1V) + 2.35 (peak distance) + 0.09 (CBI) + 0.001 (Integr. radius) + 0.002 (ablation depth). GEE, generalized estimating equation. *∗P* < 0.05 and *∗∗P* < 0.01. FS-LASIK, femtosecond laser in situ keratomileusis; TPRK, transepithelial photorefractive keratectomy; SE, spherical equivalent; K, keratometric readings in diopter; IOP, intraocular pressure; bIOP, biomechanically corrected IOP; A1L, first applanation length; A1V, applanation-1 velocity; A2L, second applanation length; A2V, applanation-2 velocity; PD, peak distance; DA, deformation amplitude; CCT, central corneal thickness; A1T, applanation-1 time; A2T, applanation-2 time; CBI, Corvis Biomechanical Index; DA ratio, DA ratio at 2.0 mm; Integr. radius, the integrated area under the radius of the inversed curvature during the concave phase; ARTh, Ambrósio's relational thickness in the horizontal profile; SP-A1, stiffness parameter at applanation 1.

## Data Availability

Access to data is restricted due to ethical concerns and patient privacy.

## Supplementary Materials

Supplementary Materials Supplementary Table S1: correlations between variables (*N* = 197).
