# Supplementary material for: Changes in Intraocular Pressure after Transepithelial Photorefractive Keratectomy and Femtosecond Laser In Situ Keratomileusis
Source: J Ophthalmol. 2021 Mar 10;2021:5592195. doi: 10.1155/2021/5592195 (PMC7972855; doi:10.1155/2021/5592195)
Supplement: Supplementary Materials — Supplementary Table S1: correlations between variables (N = 197). [file 5592195.f1.docx]

SE, spherical equivalent; A1V, applanation-1 velocity; PD, peak distance; DA, deformation amplitude; A1T, applanation-1 time; A2T, applanation-2 time; CBI, Corvis biomechanical index; Integrated radius, the integrated area under the radius of the inversed curvature during the concave phase; SP-A1, stiffness parameter at applanation 1; IOP pre-op, preoperative intraocular pressure.

| **Supplementary Table S1. Correlations between variables (N = 197)** | | | | | | | | | | |
| --- | --- | --- | --- | --- | --- | --- | --- | --- | --- | --- |
|  | SE (D) | A1V (m/s) | PD (mm) | DA (mm) | A1T (ms) | A2T (ms) | CBI | integr_Radius (mm^-1^) | SPA1 | IOP pre-op (mmHg) |
| SE | -- |  |  |  |  |  |  |  |  |  |
| A1V | .030 | -- |  |  |  |  |  |  |  |  |
| PD | .001 | .538** | -- |  |  |  |  |  |  |  |
| DA | -.002 | .787** | .725** | -- |  |  |  |  |  |  |
| A1T | -.082 | -.671** | -.702** | -.772** | -- |  |  |  |  |  |
| A2T | .142* | .734** | .515** | .713** | -.597** | -- |  |  |  |  |
| CBI | .071 | .418** | .298** | .322** | -.457** | .296** | -- |  |  |  |
| integr_Radius | -.093 | .617** | .270** | .528** | -.553** | .523** | .500** | -- |  |  |
| SPA1 | -.045 | -.768** | -.615** | -.700** | .715** | -.614** | -.621** | -.625** | -- |  |
| IOP pre-op | -.094 | -.740** | -.735** | -.801** | .889** | -.727** | -.479** | -.579** | .784** | -- |
| Depth of ablation | -.656** | -.236** | -.156* | -.222** | .362** | -.253** | -.367** | -.206** | .369** | .289** |
| Spearman's rho Coefficient. **p*<0.05, ***p*<0.01. | | | | | | | | | | |
